# A novel, tissue-selective burr for temporal bone drilling

**DOI:** 10.1007/s00405-025-09805-y

**Published:** 2025-11-25

**Authors:** Laura Ihalainen, Matti Iso-Mustajärvi, Pia Linder, Aarno Dietz

**Affiliations:** 1 Department of Otorhinolaryngology, Kuopio University Hospital, Kuopio, PO Box 100, 70029 Finland; 2 Institute of Clinical Medicine, School of Medicine, Faculty of Health Sciences, University of Eastern Finland, Kuopio, Finland; 3 Microsurgery Center of Eastern Finland, Kuopio University Hospital, Kuopio, Finland

**Keywords:** Cutting burr, Temporal bone drilling, Tissue-selective burr, Mastoidectomy

## Abstract

**Purpose:**

Temporal bone (TB) drilling is one of the most frequently performed otologic procedures. It carries the potential risk of damage to the facial nerve, sigmoid sinus, and dura. Conventional cutting burrs (CCBs) are effective at cutting bone but may easily cause soft tissue damage. A novel 5.4-mm cutting burr, the Safety Burr (SB), with a moving, self-centering ring placed around the cutting tip, was developed as a safety mechanism to prevent soft tissue injuries.

**Methods:**

The SB was compared to the 5.0 mm CCB in 20 cadaveric fresh-frozen TBs. The contact time and number of inadvertent errors were recorded. The assessments of the three otosurgeons on burr characteristics were graded on a scale of 1 (poor) to 5 (superior). Soft-tissue protection was further evaluated in three cadaveric heads.

**Results:**

No differences were observed in the mean procedure time between the SB and the CCB. The SB was perceived to be superior to the CCB in terms of soft tissue protection, absence of jumping, and precision. In the cadaveric heads, drilling on the exposed middle fossa dura or sigmoid sinus resulted in no tearing or perforation.

**Conclusion:**

The SB is a feasible alternative for TB drilling and provides soft-tissue protection. The absence of jumping contributed to superior handling and controllability of the burr. Further clinical studies are needed to comprehensively assess the tissue selectivity and its limitations in otological TB drilling.

**Supplementary Information:**

The online version contains supplementary material available at 10.1007/s00405-025-09805-y.

## Introduction

Temporal bone (TB) drilling may be required to treat conditions such as cholesteatoma, chronic otitis media, or mastoiditis, and is necessary in cochlear implant surgery [1]. Bone resorption is a frequent consequence of inflammatory processes in these conditions, which may expose delicate structures such as the sigmoid sinus, dura mater, or the facial nerve [2, 3]. Exposing these delicate structures is required especially in many lateral skull base surgical approaches. For a successful surgery, the drilling procedure must be precise to avoid damaging these structures while exposing them. In these circumstances, surgeons often have to switch to diamond burrs, which potentially prolongs the surgical time. Serious complications for the patients, as well as increased healthcare resource utilization and respective costs, are some of the consequences of the unintentional damage of soft tissue upon bone drilling in otosurgery as well as neurosurgery [4–6].

Conventional cutting burrs (CCBs) are commonly used for TB drilling because they are effective in cutting the cortical bone and underlying mastoid air cells. These cutting burrs, however, can be unsafe towards soft tissue, and damage may occur even if handled carefully [3, 7, 8]. Equally important, CCBs tend to jump when drilling bone or structures with inconsistent density or properties, like cortical bone and mastoid air cells, which is often the case in mastoid drilling [1, 3].

**Fig. 1:**
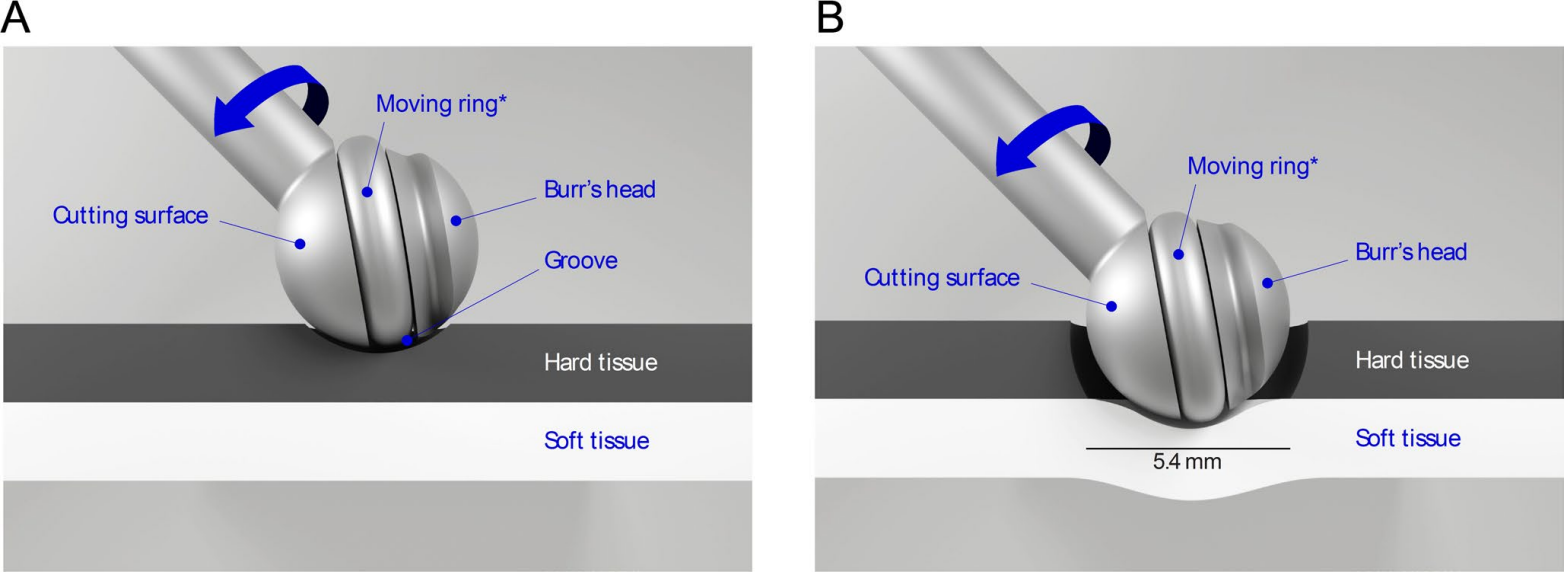
The SB operation mechanism. **(A)** Illustration of the parts and differential modes in bone cutting mode and **(B)** and in soft tissue protection mode

The Safety Burr (SB) (Halo™, Surgify Medical Oy, Helsinki, Finland) is a novel CE-marked and FDA-approved medical device [9]. This 5.4 mm tissue-selective cutting burr has earlier been used in, for example, spine surgery to provide better protection of dura mater during laminectomy. The SB integrates seamlessly with existing surgical drill consoles and features a dynamic safety-ring mechanism that forms a protective shield around the burr head, neutralizing the cutting edges upon contact with soft tissue. This technology includes a burr head with two cutting edges, a spring, and a tissue-selective safety mechanism integrated into the head. The safety mechanism consists of a pressure-controlled ring with a slightly larger diameter than the drill tip, which automatically adapts to the tissue type. The moving ring gradually moves between the two different modes. The ring retracts when pressed against hard tissues (crystalline bone) to a level lower than the cutting edges, allowing for the effective cutting and shaping of the bone (Fig. 1A). Conversely, when the burr head encounters elastic soft tissue, the ring automatically protrudes to a level higher than the cutting edges and shields the drill tip, preventing it from affecting the critical soft structures. This feature significantly reduces the damage to sensitive soft tissues in the case of accidental touch (Fig. 1B) [10].

A preclinical animal study in living sheep demonstrated the effectiveness and safety of the burr for craniotomy drilling and hemilaminectomy, preventing dural damage and bone heating [11]. Since thickness and other characteristics of the dura mater do not significantly vary with anatomic site, we theorized that the SB would provide similar soft tissue protection for the middle fossa dura in TB drilling. The aim of this study was to investigate the feasibility and drilling characteristics of the SB for mastoidectomy in fresh-frozen human TBs and compare them with the 5.0 mm CCB. Soft tissue protection was further assessed in three cadaveric heads in which we exposed the middle fossa dura and the sigmoid sinus.

## Materials and methods

### Setting and test samples

Twenty fresh-frozen human TBs were used to compare the SB (*n* = 10) to the CCB (*n* = 10). Mastoidectomies were performed by three otosurgeons (AD, LI, and MI-M) at the Microsurgery Center of Eastern Finland. TBs were drilled using either the CCB or the SB in turn and all procedures were recorded. After every drilling session, the otosurgeon completed a questionnaire on the drilling characteristics (Table 1). In addition, three cadaveric heads were drilled to assess the soft-tissue protection features of the SB.

**Table 1:** Detailed explanations of the characteristics of the burrs evaluated by the otosurgeons

Characteristics	Explanation
Efficiency of bone cutting	How fast and intensively the burr tip removes bone
Noise level	The intensity of the sound that the burr tip creates while drilling
Absence of jumping	How often the burr tip tends to jump unintentionally while drilling
Durability	How long the burr tip was evaluated to remain sharp enough for efficient drilling
Predictability	How easily the functioning the burr tip while drilling could be predicted
Precision	How easy it was to drill precise targets with the burr tip

### Surgical methods

A Stryker drill console (Stryker Corporation, Kalamazoo, Michigan, USA) was used, and the SB (5.4 mm) was operated at 37,000 rpm (maximum allowed speed by the product specification) [11]. The CCB (5.0 mm round fluted soft touch) was operated at 60 000 rpm, since this is the maximal output of the drill console and normally used in surgical operations. Mastoidectomies were performed with special attention to achieve a very thin layer of bone overlying the dura, sigmoid sinus, and the posterior ear canal wall. Completion of the mastoidectomy was defined as follows: complete exposure of the lamellae of the dura and sigmoid sinus, visualization of the incus and facial nerve, exposure of the digastric ridge and sinodural angle, and sufficient thinning of the posterior ear canal wall. In the cadaveric heads, we used only SB for each procedure. We performed an extensive mastoidectomy, exposing both the middle fossa dura and the sigmoid sinus, and drilled a complete cochlear implant bed down to the dura.

#### Outcome measures

The contact time between the burr and the bone was measured using a stopwatch. Inadvertent error was defined as a perforation of the dura, sigmoid sinus, or posterior ear canal wall. The surgeon’s assessment of the drilling characteristics was graded on a scale from 1 (poor) to 5 (superior). The assessed characteristics were the efficiency of bone cutting, soft tissue protection, noise level, jumping tendency, durability, predictability, and drilling precision (Table 1).

##### Statistical methods

Statistical analyses were performed using only the outcome measures of the TBs. We report the frequencies (n), mean values with standard deviation (SD) for normally distributed variables, and median values with interquartile ranges (IQR) for non-normally distributed variables. For group differences between the SB and the CCB, we report p-values using the T-test or Mann-Whitney U -test. All p-values were two-sided, and p-values smaller than 0.05 were considered statistically significant. As only a single comparison between the SB and the CCB was made per outcome, multiple comparison adjustment was not performed. For the statistical analyses, we used R version 4.4.3 (R Core Team, Vienna, Austria).

## Results

No statistically significant differences were found in the contact times and number of inadvertent errors (*p* = 0.69), efficiency of bone cutting (*p* = 0.14), or noise level (*p* = 0.16) between the SB and the CCB (Table 2). However, the SB was perceived as significantly superior to the CCB in terms of the absence of jumping (*p* < 0.001), durability (*p* < 0.01), predictability (*p* < 0.001), and precision (*p* < 0.001) (Table 2). With three fresh cadaveric heads, the SB was evaluated as excellent for soft tissue protection, without perforation or tearing (Supplemental Video 1). Perforation of the sigmoid sinus was created only when drilling intentionally for an extended amount of time.

**Table 2:** Characteristics of the SB compared to the CCB in 20 fresh-frozen TBs

	SB (*n* = 10)		CCB (*n* = 10)		*p*-value
	Mean	SD	Mean	SD	
Contact time (h: min: sec)	00:17:16	00:07:43	00:14:59	00:06:33	0.45
	**Median**	**IQR**	**Median**	**IQR**	***p-value***
Inadvertent errors (n)	1.0	0.75(1–1.75.75)	1.0	1.0(1–2)	0.69
Characteristics (scores)					
*Efficiency of bone cutting*	4.0	0.75(4–4.75.75)	5.0	0.75(4.25–5.25)	0.14
*Noise level*	3.5	1.75(3–4.75.75)	2.5	3.0(1–4)	0.16
*Absence of jumping*	5.0	0(5–5)	1.0	1.5(1–2.5.5)	**< 0.001**
*Durability*	5.0	0.75(4.25–5.25)	3.5	1.0(3–4)	**< 0.01**
*Predictability*	5.0	0(5–5)	2.0	0.75(2–2.75.75)	**< 0.001**
*Precision*	5.0	0(5–5)	2.0	1.0(1–2)	**< 0.001**

*CCB*, conventional cutting burr; *IQR*, interquartile range; *SB*, safety burr; *SD*, standard deviation; *TB*, temporal bone

## Discussion

In this study, the SB was used for mastoidectomies of 20 fresh-frozen human TBs, and the overall feasibility, drilling characteristics, and soft tissue protection of the SB were assessed for the first time in comparison to the CCB. Both the SB and the CCB were comparable in features such as the contact time, number of inadvertent errors, efficiency of bone cutting, and noise level, indicating that the SB falls within the range of the CCB in these characteristics. Although not statistically significant, the average occurrence of inadvertent errors was lower when drilling TBs with the SB compared to the CCB, as previously observed in a preclinical study [11]. We speculate that the improved controllability and handling of the SB may have contributed to the lower number of inadvertent errors during thinning of the bone overlying the posterior ear canal, middle fossa lamina, and sigmoid sinus.

The SB was superior in the absence of jumping, durability, predictability, and precision, features that enhance the safety of the operation, and drilling experience. We observed no tendency for jumping when using the SB; thus, it provided a superior drilling experience with enhanced precision and predictability compared to the CCB. The SB achieves optimal performance at approximately 50% of its maximum rotational speed. This is because the cutting edges are specifically designed to maintain efficiency and controllability at low speeds. Features such as rake angle, sharpness, and other cutting parameters are optimized to enhance performance even under these conditions. Thus, unlike CCBs, the SB can operate effectively at lower speeds, minimizing chattering without losing controllability or cutting efficiency [11].

To analyze the soft tissue protection capabilities in more detail, we performed additional testing on three fresh cadaveric heads with the SB. In all cases, extensive mastoidectomy was completed, during which the tegmen dura and sigmoid sinus were safely and completely exposed without causing lacerations of these structures. The sigmoid sinus was eventually intentionally damaged; however, excessive drilling of the vein wall was required to cause perforation. It is important to note that with the SB to ensure adequate bone cutting, surgeons need to adjust their drilling technique using gentle pressure, similar to drilling with a coarse diamond burr [12]. In addition, the surgeon can adjust the speed of the SB according to the need in different stages of the surgery, e.g., the surgeon can remove bone at a lower rate close to sensitive tissues. Furthermore, a lower rotational speed reduces the heating of the drill and adjacent tissue.

The main limitation is that we did not assess facial nerve protection because of the large burr size in relation to the narrow diameter of the fallopian channel. However, we will assess the facial nerve protection in consecutive preclinical studies with smaller burrs. Another limitation is that the surgeons could not be blinded to the burr type given that drilling was performed under visual control with an operating microscope.

## Conclusion

The SB was found to be feasible for mastoidectomy and demonstrated enhanced drilling characteristics, contributing to greater predictability and precision in temporal bone (TB) drilling. In particular, extended mastoidectomy could be performed efficiently with safe exposure of the sigmoid sinus and middle fossa dura. Moreover, it provides superior levels of control and precision through reduced chattering—sudden, uncontrollable, and potentially dangerous jumping of the burr—compared to conventional burrs, ensuring accuracy and stability in bone removal procedures. Thus, with proper training, the SB could provide safer TB drilling conditions. Additional research is needed to comprehensively assess the features and limitations of this device in otologic surgeries. Continued development should focus on producing smaller burrs to further expand the clinical applications of the SB.

## Supplementary Information

Below is the link to the electronic supplementary material.

Supplementary Material 1

## Data Availability

Data obtained from this study will not be made publicly available, but will be available for collaborative sharing after the main results of the study have been published.

## References

[1] Isaacson B (2018) Anatomy and surgical approach of the ear and temporal bone. Head Neck Pathol 12(3):321–327. 10.1007/s12105-018-0926-2 30069845 10.1007/s12105-018-0926-2 PMC6081290

[2] Wiatr A, Strek P, Wiatr M (2021) Patterns of bone damage in patients with chronic middle ear inflammation. Ear Nose Throat J 100(10):NP438–NP443. 10.1177/0145561320924144 32397813 10.1177/0145561320924144

[3] Luers JC, Hüttenbrink KB (2016) Surgical anatomy and pathology of the middle ear. J Anat 228(2):338–353. 10.1111/joa.12389 26482007 10.1111/joa.12389 PMC4718166

[4] Strömqvist F, Sigmundsson FG, Strömqvist B, Jönsson B, Karlsson MK (2019) Incidental durotomy in degenerative lumbar spine surgery – a register study of 64,431 operations. Spine Journal 19(4):624–630. 10.1016/j.spinee.2018.08.012 10.1016/j.spinee.2018.08.012 30172899

[5] Puvanesarajah V, Hassanzadeh H (2017) The true cost of a dural tear. Spine 42(10):770–776. 10.1097/BRS.0000000000001895 27584677 10.1097/BRS.0000000000001895

[6] Milton R, Kalanjiyam GP, Shetty RSAP, Kanna RM (2023) Dural injury following elective spine surgery – a prospective analysis of risk factors, management and complications. J Clin Orthop Trauma 41:102172. 10.1016/j.jcot.2023.102172 37483912 10.1016/j.jcot.2023.102172 PMC10362543

[7] Kennedy K, Lin J Mastoidectomy, StatPearls, StatPearls Publishing. Accessed: May 15, 2024. [Online]. Available: https://www.ncbi.nlm.nih.gov/books/NBK559153/

[8] Papavero L, Engler N, Kothe R (2015) Incidental durotomy in spine surgery: first aid in ten steps. Eur Spine J 24(9):2077–2084. 10.1007/s00586-015-3837-x 25735610 10.1007/s00586-015-3837-x

[9] Food and Drug Administration 510(k) Premarket Notification. Accessed: Nov. 14, 2024. [Online]. Available: https://www.accessdata.fda.gov/scripts/cdrh/cfdocs/cfpmn/pmn.cfm?ID=K232684

[10] Sippola V, Najafi Haeri S. A surgical burr. Accessed: Nov. 14, 2024. [Online]. Available: https://data.epo.org/publication-server/rest/v1.0/publication-dates/20200311/patents/EP3525691NWB1/document.pdf

[11] Vartiainen N, Niemelä M, Pälvimäki E-P (2025) Evaluation of a novel high speed burr tip for safe and efficient bone surgery in a sheep model. Sci Rep 15(1):16864. 10.1038/s41598-025-01769-7 40374750 10.1038/s41598-025-01769-7 PMC12081626

[12] Dillon NP, Kratchman LB, Dietrich MS, Labadie RF, Webster RJ, Withrow TJ (2013) An experimental evaluation of the force requirements for robotic mastoidectomy. Otol Neurotol. 10.1097/MAO.0b013e318291c76b 23787968 10.1097/MAO.0b013e318291c76b PMC3761064

